# Social influences on mental health help-seeking after interpersonal traumatization: a qualitative analysis

**DOI:** 10.1186/1471-2458-10-634

**Published:** 2010-10-22

**Authors:** Viola Schreiber, Andreas Maercker, Babette Renneberg

**Affiliations:** 1 University of Zurich, Psychopathology and Clinical Intervention, Binzmühlestraße, Zürich, Switzerland; 2 Freie Universität Berlin, Department of Educational Science and Psychology, Habelschwerdter Allee 45, 14195 Berlin, Germany

## Abstract

**Background:**

Despite frequent and serious mental health problems after interpersonal traumatization, only a fraction of those affected by interpersonal violence seek formal help after the event. Reasons for this mismatch can be found in the individual help-seeking process but also in the individual's social environment. These social factors are explored based on a model describing the survivor's help-seeking process.

**Method:**

Survivors of interpersonal traumatization and professionals providing help for this population were asked about factors influencing the ease of seeking and receiving professional help after interpersonal traumatization. A deductive and inductive content analysis of the experiences of 43 survivors of interpersonal traumatization and 16 professionals providing help for this population was carried out.

**Results:**

The analysis suggested a clear distinction of an individual and a social system level of influencing variables. At the system level three main factors were identified: factors of the help-system, dominant attitudes in society and public knowledge about traumatization and available help.

**Conclusions:**

The results confirmed a complex interaction of variables on the individual and system level in the help-seeking process. The system level affects the individual's help-seeking through multiple pathways, especially through the individual's representation of the traumatization, through the reactions of the individual's social network and through barriers the individual perceives or experiences in the formal help-system.

## Background

Interpersonal traumatization like physical or sexual assault is a common problem in many societies [1-5]. A high prevalence of mental health problems is observed in survivors of such traumatization; first of all post-traumatic stress disorder (PTSD) but also major depression, anxiety disorders, obsessive-compulsive disorder, alcohol and substance abuse, somatization, and sexual dysfunction [6-10]. These consequences of traumatization are associated with functional impairment, a significantly reduced quality of life and high rates of physical health problems [11-16]. Nevertheless, only a fraction of the traumatized individuals seem to seek psychosocial help, hence a significant proportion of survivors of trauma do not receive the indicated care [17-22].

**Figure 1:**
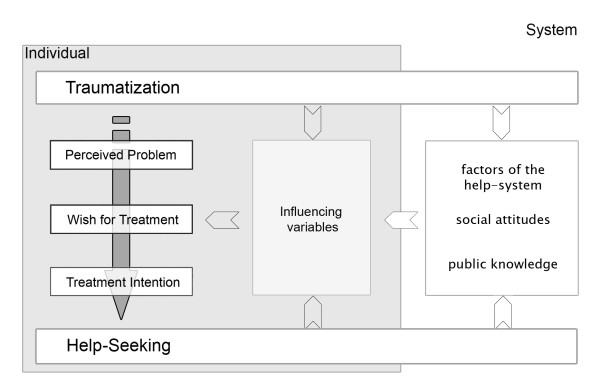
**The structure of the integrative model of help-seeking**.

Reasons for this mismatch have been explored in studies with survivors of interpersonal traumatization and have been integrated in a model describing why survivors of interpersonal violence refrain from, delay or engage in mental health help-seeking [23]. Figure 1 gives an overview of the model.

The model describes individual help-seeking as a process developing through four stages and trajectories, each influenced by specific variables. In the first trajectory the individual translates the interpersonal traumatization in a perceived problem composed of the experience of any symptoms, worry, suffering, and impairment, and the cognitive representation of the trauma and its sequelae. In the second trajectory the perceived problem may give rise to a wish for treatment, representing the felt need for professional help (like psychotherapy or counseling) in order to cope with the perceived problem. On the basis of this wish a treatment intention can be formed in the third trajectory. It is the result of a deliberative assessment of the desirability and feasibility of the wish for treatment. The last trajectory comprises any planning and action based on the intention and ends in observable help-seeking. As the influencing variables will not be subject of this paper, they will not be elaborated here, for more information please refer to [23].

In this study data from free answers to two open-ended research questions were analyzed to revise the model's structure. This paper presents the study results on the influences of the social environment on the individual help-seeking process.

## Method

### Participants

A traumatized sample was drawn from the participants of an online survey on help-seeking after interpersonal traumatization conducted by the authors. The participants were recruited through a variety of sources by informing the sources' users about the study and asking them to participate online. The majority came from internet self-help forums (60%), the remainder from other sources on the internet (like other forums and content sites; 23%), and non-internet sources (like physician's offices; 17%). None of the participants received payment for his or her participation, but we provided feedback if wished and the open-ended research questions of the study were included for all participants with an interpersonal trauma (128 women and 15 men) with the request to email a written answer. To ensure anonymity, answers were saved without email address or name and without connection to the existing data from the online questionnaire.

In a second step a sample of professionals working in the field was contacted through the mailing list of a crisis line (Telefonseelsorge), a victim assistance agency for crime victims (Weisser Ring), the National Association of Women's Counseling and Rape Crisis Programs **(**Bundesverband Frauenberatungsstellen und Frauennotrufe) and a psychotherapy newsgroup (Deutschsprachiges Psychotherapie-Forum im Internet DPI e.V.).

### Procedure

Two open-ended research questions were emailed to the potential participants. For the traumatized sample the questions were: "What would have made it easier for you to seek professional help after the event?" and "What do you think would make it easier for others to do so?" For the professional sample the questions were: "From your own experience: What do you think would make it easier for somebody who experienced a traumatic event (especially interpersonal violence) to seek and receive professional help afterwards?" and "What would have to change (e.g. in the help-system or in society) to reduce barriers between those seeking and those providing help - and how could that be achieved?" These questions were formulated with the aim to elicit promoters of help-seeking. Nevertheless, they were frequently answered by - often explicitly - naming barriers the individuals encountered or perceived in help-seeking. Given this valuable data we decided to analyse the answers in regard of positive as well as negative influences on help-seeking.

The study was conducted following the ethical standards of the German and Swiss Psychological Associations. According to the rules of procedure of the Swiss and the German Psychological Society a formal approval of the project was not necessary as the standards of voluntariness, confidentiality and inoffensiveness were closely followed.

### Data analysis

The qualitative analysis of the answers combined directed, deductive content analysis and conventional, inductive content analysis [24,25]. For the directed analysis aspects of analysis were defined, and a set of main- and sub-categories were developed based on the integrative model of mental health help-seeking after traumatization [23,26]. The model describes the intrapsychic help-seeking process and the influencing variables hampering or promoting help-seeking. Consequently, three aspects of analysis were chosen: variables of the intrapsychic process and influencing variables being either barriers to help-seeking or promoters of help-seeking. For each of these variables we formulated a definition, coding rules and examples for the variable's main category, and if necessary, for all sub-categories. Wherever possible, aspects hampering (barriers) or promoting (promoters) help-seeking were differentiated.

The resulting coding agenda was assessed for clarity by a co-worker who knew the model but was not too familiar with it. Where necessary, the coding agenda was revised.

In the second step all respondents' answers were read and the first half was coded using the coding agenda. During this process new categories or sub-categories were developed whenever those deductively developed were insufficient, and consequently the coding agenda was revised again. In the third step the first author coded all answers (including those already coded) using this revised coding agenda. During the coding process problems or 'not-yet-understoods' were noted down as a later starting point of further analysis [27]. Subsequently all answers were recoded by the first author and the co-worker respectively without knowledge of the other's coding. The recoding-reliability and the intercoder-reliability were already satisfying at this point (Cohens κ .86; Cohens κ .76) [25]. Nevertheless, all 'not-yet-understoods' were discussed together with those noted earlier and the coding agenda was revised and the coding adapted. In the fourth step the model was re- and co-constructed based on the integrative model and the categorized quotes.

## Results

### Respondents

Of all 143 survivors of interpersonal traumatization (T) 43 (30%) answered the questions. The traumata included (continuous) partner violence, physical and sexual abuse during childhood or sexual assault. Sixteen professionals (P) answered the emailed request. Four answers came from staff of the crisis line, four from local branches of the victim assistance agency for crime victims, five from members of the National Association of Women's Counseling and Rape Crisis Programs and three via the psychotherapy newsgroup.

### The answers

Written answers differed in length - ranging from a few sentences to a whole page. A typical answer was about 200-300 words long (mean 262, range 43-1074). The answers of the respondents and - to a lesser extent - the professionals contained statements at two levels of abstraction. They described individual experiences as well as subjective theories about what hampered or promoted psychosocial help-seeking. Only a few respondents made a difference between factors which would have helped themselves and those they thought to be helpful for others. A rich set of quotes (about 200) was coded and analyzed.

### Barriers and promoters affecting help-seeking after interpersonal violence

Data analysis supported the assumption that help-seeking after interpersonal violence is affected by a multitude of individual, structural and social factors. These factors can be assigned either to the individual process of help-seeking itself, or to the system in which this process is taking place. The influencing variables at the system level include (1) the factors of the system providing the help, (2) the dominant attitudes in society regarding interpersonal traumatization, mental health problems and help-seeking, and (3) public knowledge about traumatization (about traumata, their consequences and sources of help).

One traumatized respondent captured all three system variables in her answer:

Help-seeking would have been easier for me if the skepticism which is unfortunately still prevalent in society and this negative touch had not been given or at least not to this extent. […] Only by chance and by a lot of initiative did I learn that something like trauma-therapy exists… Looking back I wished that someone during the prior long and hard time had told me before, so I would have saved nearly a whole precious year! […] In my opinion it is crucial that this problem and topic is much more openly discussed and made accessible […] especially at prominent places like physicians' offices, hospitals or rehab clinics […]. (T26)

#### Factors of the help-system

The factors of the help-system dominated the answers regarding the implementation of the intention to seek help (mentioned by 47.4% of the respondents), but they also turned out to be of special relevance on the other trajectories too (mentioned from 8.5% to 20.3% of the respondents). They were seen to influence the perception of the problem as well as influencing variables on all other trajectories in the model like interpersonal trust, the attitude to help-seeking or the assessment of the feasibility of help-seeking. Table 1 gives an overview of the relevant aspects of the factors of the help-system for the model's four trajectories.

**Table 1:** Relevant aspects of the factors of the help-system for the model's four trajectories

Trajectory	Respondents' descriptions	traumatized		professionals	
		%	N	%	N
1. Trajectory: Traumatization to problem perception	Knowledge about traumatization; lack of time, care or sensitivity *It didn't even occur to the physicians and therapists with whom I dealt with that anything like a traumatization exists, even though I touched on the subject several times. [...] It would be helpful, if as many people as possible - especially in the mental health field - would know what effect traumatization has and would be sensitized for this problem [...] (T30)*	7,0	3	12,5	2
2. Trajectory: Problem perception to wish for treatment	Long waiting times; negative experiences with the help-system versus profiles of help agencies, which give clear information about the target group, about its offers, staff, and access to the service and a more proactive approach. *All my prior therapists [...] always wanted to find problems in my childhood which did not exist or spread nonsense l[...] How is one supposed to build up trust given that. Next week I take up therapy for the last time, if they talk with me again as if I was retarded, then there's no point in that. (T35)*	18,6	8	25	4
3. Trajectory: Wish for treatment to treatment intention	Tight schedule; long waiting times; lack of information versus low threshold services; exemption from charges, easy to reach, promptly available and proactive. *It is hell to "just" tell somebody about it - in the 5 minutes a general practicioner dedicates for a patient. (T1)*	18,6	8	31,25	5
4. Trajectory: Treatment intention to help-seeking	Shortage of resources; difficult access to services due to formalities; lack of offerings for specific problems; insufficient knowledge about or sensitivity to the problem; minimization of the problem; lack of referral and networking; little (pro)active support *There are vanishingly few offers of help, the waiting time is unreasonablely long. (P15) My past experience showed that traumata were preferably trivialized [by professionals]. (P7)*	39,5	17	68,75	11

Each respondent mentioning an influencing variable was counted once, even if his/her statements regarding the variable addressed different aspects

The main issues in the answers were: (1) shortage of resources: lack of therapists in the local area, lack of therapy places, long waiting lists, tight consultation time or restricted counseling hours which do not accommodate disclosure, (2) difficult access to services: e.g. due to formalities, (3) lack of offers for specific problems (e.g. for complex comorbidity or underage victims), (4) helpers' insufficient knowledge about or sensitivity to the problem, (5) negative reactions: blaming, minimization, unresponsiveness, (6) lack of (pro)activity: insufficient referral, networking or support, and (7) insufficient provision of information about the services by the providers. This last issue included information about specialized treatment but also about the setting pertaining to autonomy, trust, dynamics of relationships and fear of confrontation. The traumatized respondents primarily described barriers resulting from the issues (1), (4), (5) and (7) while the professionals focused on the issues (1) and (6).

All these factors interacted with the loss of resources resulting from the trauma.

For me it is the worst of all that I scream for help but nobody takes it seriously. That I have to struggle with public authorities in order to receive help when I already have my hands full with my own problems. Because the psychiatrist doesn't consider it serious enough, I am not allowed a hospital stay. Waiting time for a place in therapy is about two years. Those who can't deal with it themselves fall by the wayside. (T9)

For many traumatized respondents the first contact with the help-system was with a general practitioner or with non-clinical helpers like teachers and social workers from other areas and they expressed the wish that (primary) care providers should be more sensitive to and knowledgeable about the issue of interpersonal traumatization, actively follow up any suspicion of interpersonal violence, thoroughly assess mental health problems and traumatization and correctly diagnose its consequences. Failing to do so was experienced as insensitivity and a lack of competence by the traumatized respondents.

The structural barrier most frequently mentioned by both groups was long waiting times for therapy or counseling. A traumatized respondent described her experience with the help-system as follows:

No, no place in therapy vacant, no waiting list, no referral to another therapist. I'm completely down. Sad and disappointed. Though I know of course that it is a stroke of luck if it works out on the first try. But to have achieved so absolutely nothing is very frustrating for me. This feeling of not getting any help, when I've already come this far and asked for it (which took a lot of effort for me to do!). (T41)

Furthermore many respondents (27.9% of the traumatized, n = 12; 37.5% of the professionals, n = 6) described how they/a traumatized person contacted a help-provider and received some form of help but not the help they needed. Such negative experiences with help-seeking may pose a barrier to help-seeking as illustrated in the following quote:

Each time a traumatized person has encouraged himself to confide in someone and has had the experience that this person can't help him to understand himself and what happened to him (and to put back straight what is upside down), some part of the context of this encounter turns into a 'barrier' for him, which can hamper seeking help. (P12)

Here the main issues were: (1) insufficient knowledge about or sensitivity to the problem, (2) minimization of the problem, (3) mismatch between help offered and needed, (3) (feared) loss of autonomy, (4) insufficient resilience and mental hygiene of the helpers, (5) lack of referral and networking, and (6) tight consultation time. A traumatized respondent's statement wrapped it up:

It is not a question of getting ANY help, it has to be the appropriate help. (T23)

The respondents pointed out the importance of low threshold services, i.e. services without charges that are easy to reach (via consultation hours, location, phone or email), promptly available and proactive, as well as specific training of the help-providers in care for victims of traumatization to reduce barriers in the actual process of help-seeking.

#### Attitudes in society

Public attitudes about interpersonal violence and mental health play an important part in shaping the social environment in which the survivors and the help-providers are embedded.

This system variable has multiple pathways on the process of help-seeking - see Table 2 for an overview.

**Table 2:** Relevant aspects of the social attitudes for the model's four trajectories

Trajectory	Respondents' descriptions	traumatized		professionals	
		%	N	%	N
1. Trajectory: Traumatization to problem perception	Taboos/a veil of silence; dismissing some forms of interpersonal violence as peccadilloes; attribution of accountability for problems in relationships to the women; stigmatization of mental health issues; toughness and violence as an integral part of some subcultures and association of victimization with weakness. *As a consequence, trauma survivors perceive themselves as suffering somehow, just not mentally. That would be a sign of weakness. (P11)*	7,0	3	18,75	3
2. Trajectory: Problem perception to wish for treatment	Taboos/veil of silence, negative attitude to mental health problems/counseling/therapy/victimization versus tolerance for the problem and the need for help. *Help-seeking would have been easier for me if the skepticism which is unfortunately still prevalent in society and this negative touch had not been given or at least not to this extent. (T26)*	9,3	4	43,75	7
3. Trajectory: Wish for treatment to treatment intention	Consideration of/for the victim and a clear position toward the responsibility of the perpetrator. *Many women come pretty late - because in our society women are held responsible for the relationship. This leads them to lay the blame on themselves, to think it is their fault: "if only I would have been a little more considerate my husband would not have gone apeshit", "if only I would have made sure that the children are calm..." (P16)*	4,7	2	18,75	3
4. Trajectory: Treatment intention to help-seeking	The *attitudes in society *were seen to affect the help-system through the importance attached to the problem of interpersonal violence, its victims and their support-system. *Higher significance of traumatization in society - more awareness of the problem, more budget. (P2)*	7,0	3	6,25	1

Each respondent mentioning an influencing variable was counted once, even if his/her statements regarding the variable addressed different aspects

In the study, 27.1% of the respondents mentioned social attitudes. They ascribed social attitudes relevance for all social variables in the help-seeking process (like social support), for the transfer of knowledge and even the wish to talk about the experiences. In addition social attitudes were seen to affect the help-system through the relevance ascribed to the problem of interpersonal violence, its victims, and their support system. An especially close relationship was described between the individual's attitude to help-seeking and the system level variable social attitudes. The integrative model assumes that - at the individual level - a stoic attitude as well as fear of stigmatization is relevant for a wish for treatment to emerge. Both aspects emerged in our respondents' answers while most described a fear of stigmatization in one or another form. The fear of the individual of being stigmatized is associated with the culturally transported negative attitude to mental health problems, counseling/therapy and victimization. This role of stigma in society was more frequently described by the professionals. They described how the social attitude fuels the fear of being stigmatized by others, but it also strongly influences the individual's own attitude. This can lead to self-labeling or self-devaluation. Seeking help for a mental health problem was related to 'being mad' or to weakness. Other respondents wrote how they felt that they had to cope by themselves or that they were afraid of becoming too much determined from others.

Help-seeking would have been easier for me if […] this negative touch was not given or at least not to this extent. In my case it was certainly further complicated by my temperament or character respectively and by the fact that I usually wangled everything on my own. As I say - true to the maxim: "ME, going to a psychiatrist/psychologist? ME? Rubbish! I'm not out of my head?!! (T26)

#### Knowledge

In the traumatized respondents' answers individual knowledge about traumatization turned out to be of prominent relevance. The integrative model proposed that knowledge about traumatization is important for the accurate representation of the traumatization. This was supported by the respondents, who saw insufficient knowledge about interpersonal traumatization and its consequences as a barrier to accurate problem perception while the availability of knowledge promoted it. But more than the integrative model all respondents emphasized the relevance of public knowledge for all trajectories of the process. The relevant aspects of this system variable are summarized in Table 3.

**Table 3:** Relevant aspects of the public knowledge for the model's four trajectories

Trajectory	Respondents' descriptions	traumatized		professionals	
		%	N	%	N
1. Trajectory: Traumatization to problem perception	Knowledge about traumatization and its consequences in general but also about interpersonal violence in specifics. *Even today the people in my surroundings don't have the faintest idea what it means to be traumatized. They simply can't imagine it and are/were therefore not helpful. (T43)*	20,9	9	6,25	1
2. Trajectory: Problem perception to wish for treatment	Knowledge about traumatization, knowledge about the chances and possibilities of professional help for traumatization, what help is available (by law, by the police, by shelters, counseling or therapy) and for whom. *Professional helpers and laymen who are not familiar with the subject are at times not very helpful and make insensitive, degrading comments. (P4)*	2,3	1	31,25	5
3. Trajectory: Wish for treatment to treatment intention	Knowledge about the prevalence of victimization through interpersonal violence; knowledge about available forms of help, the access to it and what happens there with regard to methods, content, professional secrecy and autonomy *Second... better information! [...] about the different forms of therapy, the possibilities of financing by health insurance, how can I find the appropriate therapist for me etc. In self-help internet forums [...] you regularly find threads with the question "how can I find a therapist". (T1)*	7,0	3	12,5	2
4. Trajectory: Treatment intention to help-seeking	Knowledge where to find and how to access available forms of help *More information about who offers help and about places to go (P16)*	4,7	2	12,5	2

Each respondent mentioning an influencing variable was counted once, even if his/her statements regarding the variable addressed different aspect

Multiple processes mediate between this public knowledge and the individual help-seeking process. If knowledge is 'public' it is more likely to be part of the general knowledge an individual already possesses pre-trauma. In addition, public knowledge influences how the social environment represents and reacts to the traumatization. Furthermore, the respondents made it clear that the active role survivors of traumatization take in help-seeking also includes actively seeking knowledge: on the internet, in forums, in books or in conversations. Depending on their position in the process they sought different knowledge. On the first trajectory they sought knowledge about their problem in order to make sense of their experiences (mentioned by 16.9% of the respondents). On the following trajectories information about available forms of help and access to it became relevant, e.g. for the assessment of the feasibility of help-seeking (mentioned by 6.8 to10.2% of the respondents). The analysis left the question unanswered as to what triggers and maintains such information seeking. Possibly this depends on the antecedent variable of the intrapsychic process, e.g. the strength of the wish for treatment, and on the characteristics of the person and her/his environment, e.g. availability of internet access.

## Discussion

Sixteen professional help-providers and 43 respondents who experienced an interpersonal traumatization answered a question about factors influencing the ease of seeking and receiving professional help after interpersonal traumatization. The answers were qualitatively analyzed following a deductive approach driven by the integrative model of help-seeking after interpersonal traumatization supplemented by inductive analysis [23-25]. The analysis confirmed that help-seeking is a complex process, combining individual and system-level variables in which the effects of the trauma, the social environment and factors of the help-system interact.

The results of the qualitative analysis suggested completing the picture of the individual help-seeking process through a stronger consideration of the system-level variables. While the original integrative model acknowledged the relevance of the system level, it was not further elaborated. Based on the study results, the model was revised and now includes three system-level variables: factors of the help-system, dominant attitudes in society and public knowledge.

This stronger emphasis on the system level is in line with an ecological framework describing the interdependence of individuals and their social environment. Individual responses to traumatic events can thus be conceptualized as the result of complex interactions among individuals, events and sociocultural context. The sociocultural context provides a source of meaning, appraisal and understanding of the event for the victim and for family, friends and help-providers [28,29]. The interaction of individual and context influences the psychological response, coping, the availability of informal sources of help as well as access to and comfort with the professional help-system [30-33]. This view was consistently mirrored in the answers of traumatized as well as professional respondents who described multiple interactions on all of the model's trajectories and for most of the influencing variables.

A comparison between the answers of traumatized respondents and professionals showed that system-level variables were covered to a relatively higher proportion in the professionals' answers - while the contrary was true for the individual-level variables. This is not surprising given the different point of view and hence different insight in the individual process.

Factors of the help-system were dominant in our respondents' answers. This is contradictory to findings by other studies of mental health help-seeking where factors of the help-system seemed to play only a secondary role [34,35]. Meltzer and colleagues asked for reasons for deciding not to seek help or for turning down help at a single instance,reduced opportunities to seek help due to factors of the help-system were not assessed [34]. Most of their respondents gave only one reason and as the effect of factors of the help-system often is mediated it might have been understated. Thompson and colleagues used a forced choice format and the only structural factor was cost (couldn't afford help) [35]. In contrast, we asked in the present study "what would make it easier to seek help?" and most respondents mentioned multiple variables. This procedure might have elicited the mentioning of factors of the help-system for three reasons: (1) factors of the help-system often occurred as second-order variables influencing the reasons on the individual level (like problem perception), (2) factors of the help-system were more important further down the help-seeking process, and (3) factors of the help-system often became barriers for further help-seeking during or after first contact with the system providing the help. In fact, in studies which explicitly asked for 'not seeking a clinician's help after a traumatic event despite wanting to' factors of the help-system were frequently mentioned - ranging from perceived lack of time, knowledge or interest of the clinician and the clinician 'not asking', to the wish for more attentiveness, activity and referring on the side of the clinician [36-38]. From the complementary point of view a majority of primary care physicians felt inadequately informed and prepared regarding domestic violence or lacked knowledge in regard of PTSD [39-41]. Our respondents also described such lack of knowledge as a barrier in the contact to the primary health-care setting which is very likely to have a special position in regard of identifying traumatization and promoting problem perception given the prominent increase in health care but not in mental health care in survivors of interpersonal violence [35,42-45]. Our respondents call for more information on the issue in basic and advanced training, continuing education, educational outreach or in-service training, congresses or supervision is shared in the literature [39,40,46,47]. Both sources perceived a need for knowledge about interpersonal traumatization but also about management protocols and referral agencies [39,40,44,48,49]. But an effective intervention cannot restrict itself on information transfer. It has to be based on an assessment of potential barriers to change, and be sufficiently comprehensive to reduce these potential barriers [47]. These include not only lack of training in the necessary skills but also the effect of stigma, fear of consequences or motivation for screening, and therefore, training has to address social attitudes as well [40,41,47,50,51].

Beyond diagnosis position the factors of the help-system were repeatedly mentioned in association with dissatisfying experiences with the help-system. Such dissatisfaction with the care received has also been identified as a problem in the literature on health care of traumatized individuals [52]. Only a fraction of individuals with a traumatic experience or PTSD seem to receive or be referred to appropriate treatment [40,42,50,53]. One of the reasons for this shortfall is seen in the shortage of resources in the help-system (e.g. time constraints, lack of referral networks) [40,41,47,50,51]. For our respondents this was the barrier in the help-system mentioned most frequently. They felt that more time is needed for identification and management of the problem and especially for immediate crisis intervention. They highlighted the need for more therapists and counsellors qualified for post-trauma care, a contingent of therapy places available for immediate response and funding for specific services.

Several limitations resulted from the study design, especially from securing as much anonymity as possible. The sample was self-selected and the answers of the traumatized respondents were not linked to the existent demographic data. Consequently, the characteristics of the sample are unknown, and it is likely not to be representative of all traumatized individuals. Furthermore, the sample was rather small and the qualitative analysis did not allow for decisions about the "true" relevancy of each variable for the help-seeking process. It is possible that the dominance of factors of the help-system in the respondents' answers corresponds to their dominance as a barrier. But this dominance might also have been elicited by the wording of the question. In addition, not mentioning a variable in an open answer does not necessary mean that this variable is irrelevant to the respondent. These considerations limited the interpretation of the frequencies of mentioning.

## Conclusions

The answers of our respondents revealed interacting influences on help-seeking on the individual and system level. The underlying model was changed accordingly to comprise a clear distinction of an individual and system level of influencing variables. On the system level factors of the help-system (like difficult access), dominant attitudes in society (like blaming of the victim) and public knowledge about traumatization, its consequences and available help were included in the model. The refined model highlights the role of system level variables for individual help-seeking and can inform further research as well as the development of interventions targeting the influencing social factors.

## Competing interests

The authors declare that they have no competing interests.

## Authors' contributions

VS designed and conducted the study, analysed the data and drafted the manuscript. BR and AM supervised the project, and edited the manuscript. All authors read and approved the final manuscript.

## Pre-publication history

The pre-publication history for this paper can be accessed here:

http://www.biomedcentral.com/1471-2458/10/634/prepub
